# Report of the Committee on Practical Medicine and Epidemics

**Published:** 1866-09

**Authors:** H. Noble

**Affiliations:** Heyworth, Ill., Chairman


### REPORT OF THE COMMITTEE ON PRACTICAL MEDICINE AND EPIDEMICS.

By H. NOBLE, M.D., of Heyworth, Ill., Chairman.

Read to the Illinois State Medical Society, June, 1866.

*Mr. President, and Gentlemen of the Illinois State Medical Society:—*

In presenting a report on Practical Medicine for the current year, I shall endeavor to give chiefly personal observations of the nature and character of diseases which have been prevalent in the sections of the State which I have, myself, been able to inspect.

The early part of the past year presented no features of particular interest. The general health was about as good as common; and there was not, to my knowledge, any epidemic of importance prevailing in the State.

In the latter part of summer, autumnal fever prevailed to considerable extent in our State, and, indeed, through the whole North-west. Bilious remittent, and intermittent fevers were common in almost every neighborhood in Illinois, but few families escaping a visit from the unwelcome disease. The type was mild, almost all cases being manageable, and yielding readily to treatment. The number of fatal cases was very small, in proportion to the number attacked. In treating this disease, I have, for many years, changed my practice of giving small doses of quinine frequently repeated, to large doses at longer intervals, say from 5 to 8 grains night and morning. I think less medicine arrests the disease when given in this way, and that there is less danger of its recurrence.

Other diseases have visited our State within the last year, some of which might be classed as epidemics; but none of them prevailed over any considerable portion of the State at any one time. Perhaps the two diseases which have attracted, most directly, the physician's attention, are cerebro-spinal meningitis and erysipelas. These diseases have been present in various localities during the past year.

The fatality attending cerebro-spinal meningitis is truly appalling. But few of the well-marked cases which have come under my observation have recovered. Dr. McVEY reported several cases of this disease at the meeting of this Society, held in Chicago, in 1864. In the summer of 1864, 8 or 9 cases occurred in DeWitt County, in this State. In 1865, the disease appeared in the prairie south of Long Point timber, in DeWitt County. There were, also, several cases in McLean County. I saw some of the first cases which occurred in DeWitt County, and I have watched the disease closely as it was possible for me to observe it. The following symptoms are generally present:—The invasion of the disease is commonly sudden, commencing with a chill, attended with severe pain in the head and back; the eyes are dull and suffused; the pulse generally frequent and feeble; the limbs ache, and the muscles are commonly sore to the touch. As the disease progresses, the pain in the head increases and concentrates at the base of the occiput; the muscles of the back of the neck become irritated and contract permanently, and, in some cases, the muscles of the back become implicated sufficiently to produce opisthotonos. The fever is not, generally, of a violent grade, but is persistent, not leaving the patient entirely, at any time. In many cases, the nervous symptoms predominate, though seldom of an active character, the patient being dull and not disposed to talk, answering questions slowly and briefly, sometimes requring a peremptory tone to secure the attention of the patient. The disease is frequently attended with rigors, which seem to be caused by morbid nervous action. Some parts of the skin of the patient is frequently "spotted," from the size of a pin-head to the size of a thumb-nail, caused, I believe, by infiltration of tissue under the cuticle. From this circumstance, I suppose, the disease derives its popular name of "spotted fever." The bowels are, commonly, regular, and the discharges appear healthy to the last stages of the disease, when not disturbed by the improper use of cathartics. The disease is generally rapid in its progress, producing, if not arrested or modified by treatment, irreparable mischief in two or three days, sometimes in much less time, as I have known death to occur in sixteen hours from the attack. The disease has chiefly attacked children and young persons, a large majority of the patients being under 12 years of age. It does not appear to be contagious, not having, under my observation, attacked more than two members of any family.

Of the treatment of this disease, I desire to say, that I consider blisters to the back of the neck, reaching up to the base of the brain, as close as possible, to be of the highest importance. Indeed, I have seen no remedy used which had any influence in checking the disease before vesication of the back of the neck was produced. In every case that I have seen, the head was drawn back and remained so until the disease yielded to treatment, or death closed the scene. This rigidity of the neck was relieved, temporarily, by blistering from the base of the brain six or eight inches down the spine. As the blister ceased to discharge, and healed, the unfavorable symptom usually returned, and would be again relieved by blistering another section of the spine immediately below the first blistered surface. Some of my patients recovered, after being blistered six or eight times. I have seen no patient recover without blistering several times. I consider blisters indispensable in the treatment of this disease, no case under my observation having recovered without them; and although many patients died that were blistered, I must say that the blisters, in every case, relieved, to some extent, the urgency of the symptoms. I would recommend, in every case of this terrible disease, a blister on the back of the neck, and reaching down between the shoulders, as the first treatment to be applied, believing it to be more efficacious than any or *all* other remedies. Opium, in large doses, and repeated frequently enough to relieve the patient of pain, is absolutely necessary. The nervous system suffers very much, and should be, as nearly as possible, relieved with opiates in some form or other. During the continuance of the disease, and in all its stages, the patient should be kept as free from pain as possible, which can be best done by the use of some preparation of opium.

Several of the cases which I saw were attacked, in the progress of the disease, with rigors, sometimes at regular intervals, but oftener without marked periodicity. These paroxysms very much resembled the chills of intermittent fever, but were not followed by the severe fever, and, at its cessation, the sweating stage. I attempted to arrest them with quinine, but did not succeed; indeed, the quinine seemed to aggravate the paroxysms and make the patient worse. In consultation with my friend Dr. McFARLAND, of Heyworth, and at his suggestion, I gave strychnia, $\frac{1}{16}$ of a grain, every four hours, and succeeded in arresting the chill completely, and my patient improved satisfactorily to convalescence. This remedy arrested the chill in every case in which I tried it, and that was a large proportion of all the cases I treated. I think nine out of ten of my cases had those nervous rigors in some stage of the disease. The duration of the disease varies from less than twenty-four hours, to two or three months, some patients getting well in eight or ten days, while others get well in six or eight weeks. Some patients die in less than twenty-four hours' sickness, others live three months and then die.

From this, it appears that the disease is sometimes attended with great violence, and at other times, or in other cases, it is slow in its progress, destroying life by a total exhaustion of the vital forces. In the outline of treatment which I have given, I have attempted to bring to the notice of the profession, the remedies which appear to be efficacious in the treatment of this disease. The minutia of detail every physician can supply for each case as it is presented to him. I have not seen enough of this disease to form a decided and fixed opinion of its nature or treatment, but I am happy to say that contact with it has lessened its terrors somewhat, and the experience of the last two years leads me to hope that cerebro-spinal meningitis will, in a short time, be comprehended and understood by the scientific physician, and yield as readily to appropriate remedies as other diseases of equal violence do, when properly treated.

There are, perhaps, but few diseases which are more fatal and give a higher per cent of mortality than the one under consideration, and there is, certainly, none in which the physician has had less cause to be satisfied with the results of treatment. But that the close scrutiny and observation of the medical profession will place it in the list of manageable diseases, I have no doubt.

Erysipelas has visited some portions of our State in an epidemic form, and in some places it appeared in a severe or malignant character. Facial erysipelas, complicated with diphtheria, was sometimes very fatal, in some instances causing death in a few hours. I noticed many cases, in which the two diseases were present at the same time, thus increasing the danger of the patient. Erysipelas, in epidemic form, has frequently visited our State, and is always attended with a high per cent of mortality. Central Illinois was visited with epidemic erysipelas about thirty years ago, which was very fatal, carrying off many victims. I noticed in that epidemic, that not one pregnant woman attacked by the disease recovered, although there was under my observation at that time many such cases. My attention was specially drawn to this fact during that epidemic, and I have been a close observer of cases of that character ever since, and I must say that I have never seen a pregnant woman attacked with epidemic erysipelas on the face that got well. I do not say that such cases are necessarily fatal, but that I have not had the good fortune to witness their recovery.

A peculiarity of erysipelas, which should always be borne in mind by the physician, is the possibility of communicating to the parturient female the virus which produces puerperal peritonitis. There is some diversity of opinion among physicians about the danger of waiting upon parturient patients, while attending on cases of erysipelas, and every physician has an undoubted right to enjoy his opinion, as to whether there be danger or not; but no physician has a right to hazard the life, or I might say sacrifice the life, of a lady, by doing what so large a portion of the medical profession believe to be dangerous and unjustifiable. Men have, individually, the right to enjoy their theories when no party is interested but themselves; but in a conflict of theories, no man has a right to *act* on a theory which, if wrong, he knows must necessarily produce the death of his patient. I speak of the disease as being fatal, because I have never known a case of puerperal peritonitis to recover, which I had good reason to believe had been produced by the virus of erysipelas.

The treatment which I have found most successful, is tonic or strengthening. In many cases, quinine is needed; and in all, the muriated tincture of iron is useful. Indeed, I may say that the tincture of iron is the main remedy, and I think it is growing in favor every year. I have treated no case of erysipelas without it for many years. The nourishment should be generous. The system needs support, and should be nourished by good digestible food.

Small-pox has prevailed to an unusual extent the past year. The facilities for its transmission afforded by our soldiers have been very great, and the disease has lost no opportunity of displaying itself. But few parts of the country have been exempt from its visits; and, although the number of cases has been very large, the per cent of mortality has been small. This disease had never prevailed to any great extent in the rural districts until within the past few years, consequently, the people knew little or nothing of the nature and character of the disease, and but few physicians in the country had had opportunities of studying it at the bedside, notwithstanding which, it has been, as far as I have seen, well treated.

I have seen a few cases which had been treated with sudorifics and stimulants, which proved fatal, but I am happy to say that I have known no educated physician to resort to that mode of treatment. The great majority of cases of this disease were modified by having been preceded by vaccinia, in which cases variola is disarmed of almost all its terrors.

Vaccination has been pretty generally practised throughout our State, and to that we are indebted for our comparative immunity from this loathsome disease. Notwithstanding the protection of vaccination is apparent to all, it is evident the people but imperfectly understand the value of the remedy, otherwise they would *all* avail themselves of its protection.

Instead of this being the case, we find in almost every community a few persons of mature age who have never been vaccinated at all, and others who have been once or twice vaccinated unsuccessfully and gave it up, supposing it would never take, because it had not on the two or three trials made. Such persons should be instructed to continue to vaccinate until it *does* take, the protection secured being ample compensation for the perseverance necessary to secure it. The degree of protection is not always properly presented to the people by the physician. Absolute and perfect protection from small-pox and varioloid is not promised by all physicians, and a feeling of insecurity and distrust is the consequence. I believe it is the duty of every physician to state explicitly to the people, that vaccination, properly performed, and repeated until the susceptibility to the disease in the system is entirely destroyed, which can only be known by revaccination producing no effect, is a perfect and unfailing protection from small-pox and its modifications. The testimony of the whole profession to that effect would remove the distrust of the public mind and make the people more anxious to avail themselves of a remedy so well adapted to their wants. Nor do I believe there is any exaggeration or overcoloring of this statement of the efficiency of the protection afforded by vaccination. Nor have I any doubts of its permanency.

The theory that teaches that the protection must be renewed occasionally, or at any specific period, is, I think, fallacious. Your reporter has been protected perfectly for over fifty years by one vaccination. Revaccination has been performed four or five times, but had no effect. I knew a family of nine children have small-pox at one time. The father and mother and mother's sister had each had vaccine disease thirty years before, and had never been revaccinated. The mother and her sister nursed the nine cases of small-pox and were not in the least affected by it. The father had varioloid in a mild form, not more than a dozen pustules showing, which dried without lymph or matter, and he had no secondary fever. I have, on five different occasions, treated small-pox in families where there were children unprotected by vaccinia, which I vaccinated from the second to the fourth day after the small-pox pustules broke out, and succeeded in every case (eleven in all) in protecting, perfectly, the children from small-pox and varioloid both, the vaccine disease not being modified or influenced by the small-pox at all, so far as I could perceive. Other gentlemen may have cases illustrating more clearly the extent of the protection afforded by vaccinia, and I hope they will give the result of their observations to the profession, for I know there is much yet to be done in the way of diffusing useful information among the people on this subject. I hope every physician recognizes the fact that it is as much his duty to prevent disease when it is possible to do so as to cure it; and I know of no disease that can be so perfectly barred from the human system as variola.

The winter, just passed, has been of remarkably uniform temperature in Central Illinois, and to that fact, I think, we may attribute our comparative exemption from pneumonia, which disease has prevailed to a limited extent only. The few cases which I saw during the winter were mild, yielding to treatment readily. But as spring advanced and the warm season approached, pneumonia of a more grave form made its appearance in different parts of the State, many of the cases terminating fatally. Since the middle of March, the most of the cases of pneumonia which I have seen required tonics early in the disease, and but few of them recovered without the exhibition of tonics or anti-periodics.

Diphtheria has not, in my observation, prevailed epidemically, though sporadic cases are not infrequent. It has generally, when not complicated with erysipelas, yielded readily to treatment. I have succeeded best with a tonic constitutional treatment, and a wash to the inflamed throat of solution of sulphate of copper, 40 grains to the ounce, applied once or twice, not more.

During the past year, I have not heard of any portion of the State being visited with epidemic dysentery. I have seen but few cases the past year, were of mild type and yielded readily to treatment.

Measles has not, to my knowledge, prevailed to any great extent, the cases which I have seen being mild and resulting favorably.

Scarlatina has visited the central portion of the State, though not, so far as I have learned, of a severe or grave form. The most of the cases which I saw were mild, running their course without complication, and leaving no unpleasant sequela.

Typhoid fever has not prevailed in Central Illinois the past season to as great an extent as it has in some previous years. I have seen some cases of mild character, which recovered satisfactorily. For a period of nineteen years, this disease has been carefully observed in McLean and adjoining counties, and during all that time the south-east part of McLean County has produced many more cases of typhoid fever than the south-west part of the same county. I am not well enough acquainted with the geological formation of the two localities to state if there be any perceptible difference, but I am positive of the fact that while one locality has been camparatively free from the disease during the period named, the other has suffered more or less every year, and many years severely. Similar differences in other contiguous localities are, I believe, not unfrequent. Typhoid fever is always a serious disease, generally taxing the resources of the physician and the vitality of the patient, each to their utmost capacity.

The treatment of this disease recommended by different members of the profession, at different times, has varied considerably. "Slight ptyalism," early in the disease, has been recommended by some; quinine, in large doses, is the favorite remedy with others; while veratria has had its advocates, zealously urging its claims to the attention of the profession. Other remedies and modes of treatment have been recommended, and each of them has been submitted to the scrutiny of the profession at the bedside, and perhaps none of them have fulfilled all that was hoped for by their advocates. Indeed, the treatment of a case of typhoid fever requires a *physician*, and not a remedy. A remedy or particular plan of treatment cannot be safely substituted for a physician, who should be always ready to modify or change the remedy or prescription to meet the varying condition of the patient. When this is done properly, the disease will be scientifically treated. I congratulate the Society on the improvement of the profession of our State in the treatment of this disease. The treatment now generally adopted, is certainly more rational, more scientific, and more satisfactory than the treatment practised ten years ago.

Great interest is now felt by the medical profession on the subject of Asiatic cholera. The approaching visitation of that awful disease, judged by the history of former epidemics, may be expected to reach us in a short time. The rapidity of its progress, and the line of its approach, may each vary from its former visits; but that the general character of the epidemic will be much changed, or the violence of the disease materially modified, we can scarcely hope.

The causes which produce cholera, if at all, are but imperfectly understood. The combination of influences are so complicated that the closest observation has not satisfactorily classified them. That a peculiar condition of the atmosphere is necessary to the epidemic character of the disease I have no doubt. This is demonstrated by the rapid spread of the disease, and, in some instances, of its more rapid decline. This was illustrated in the rapid abatement of cholera in St. Louis, when the last epidemic of cholera visited that city, the particulars of which are, doubtless remembered by every gentleman present. Whatever this peculiar condition of the atmosphere may be, it is evident that it is beyond the control of science in our present state of knowledge. No quarantine or sanitary regulation has been effectually opposed to it, and we are compelled to meet this as we do all other diseases, with the best resources of the science of medicine.

The observation of former epidemics leads us to expect satisfactory results from the judicious treatment of the first stage of the disease, the diarrhœa generally yielding to the usual remedies; but when the case has progressed to vomiting, cramps, and collapse, but few recover. The treatment to be successful, should, in all cases, be applied early; and when the diarrhœa yields, everything that would be likely to reproduce it should be carefully avoided. If we could get the people to know that, during an epidemic of cholera, what they consider an insignificant diarrhœa, is really the first stage of the terrible destroyer, and that at its first appearance, without a moment's delay, is the time they should apply to a physician, more than one-half of its victims would be saved to life and usefulness.

I, therefore, consider it to be the imperative duty of every physician to inform the people, when an epidemic of cholera is approaching, that they should live temperately. All excesses of eating or drinking should be carefully avoided. Their habits of life should be uniform and regular, that is, no excessive fatigue, followed by periods of relaxation, should be indulged. The diet should be nourishing and taken in moderation, and always at the regular periods. Cleanliness is indispensable. Everything producing an offensive odor should, if possible, be corrected. Free ventilation of all parts of the house should be secured. At the first appearance of diarrhœa, consult your physician. A few hours delay may change an apparently simple diarrhœa into a fatal case of cholera. Depend on no specifics or cholera doctors. No skill or science known to man can insure uniform success in cases of fully developed cholera. The only safety is in the proper treatment of the first or diarrhœa stage.

When the people understand and strictly follow these directions, cholera will be disarmed of the chief part of its terrors.

